# Isolated Herniation of Gallbladder Through Diaphragmatic Defect Following Hepatic Microwave Ablation

**DOI:** 10.31486/toj.25.0071

**Published:** 2026

**Authors:** Ivan Buitrago, Jordan Spring, William C. Conway

**Affiliations:** Department of Surgical Oncology, Ridley-Tree Cancer Center, Santa Barbara, CA

**Keywords:** *Diagnostic imaging*, *instrumentation*, *surgery*

## Abstract

**Background:**

Thermal ablation is commonly used to control metastatic hepatic lesions. The documented overall major complication rate is low (4.1%), with a lower incidence of damage to organs (<0.7%). Diaphragmatic injuries resulting from thermal ablation can cause pain and pleural effusions and, rarely, herniation of intra-abdominal contents into the thoracic cavity.

**Case Report:**

We present a case of isolated gallbladder herniation through a diaphragmatic defect after microwave ablation. The patient's complication was corrected surgically with robotic reduction of the herniated gallbladder, closure of the diaphragmatic defect, and cholecystectomy.

**Conclusion:**

To our knowledge, diaphragmatic hernia causing herniation of the gallbladder has not been previously reported. Our patient's diaphragmatic defect became symptomatic after a delay of 2 years. Such a delay is common, and practitioners should be aware of delayed symptoms after ablation as early detection can improve patient outcomes.

## INTRODUCTION

Thermal ablation, which is commonly used for local control of hepatic metastatic lesions, is considered a safe procedure with low complication rates. A 2014 systematic review of complications after liver tumor ablation reported a major complication (any complication that caused symptoms for more than 1 week, delayed discharge, was life threatening, or led to morbidity or mortality) rate of 4.1% and a minor complication (any complication that did not meet the major complication criteria) rate of 5.9%.^1^ Fonseca et al examined major complications of hepatic tumor ablations by type of complication and found a low rate (0.5%-0.7%) of reported visceral damage (injuries to the colon, stomach, gallbladder, kidney, diaphragm, abdominal wall, or small bowel).^2^ Diaphragmatic injuries associated with thermal ablation are uncommon; however, case reports document diaphragmatic defects causing persistent pleural effusions requiring surgical repair,^3^ herniation of the large intestine,^4^ herniation of the small intestine,^5,6^ and herniation of the liver^7^ (Table). We present a case of diaphragmatic hernia causing herniation of the gallbladder.

**Table.** Summary of Case Reports Documenting Diaphragmatic Defects After Liver Tumor Ablation

Study	Liver Segment(s) Ablated	Herniated Organ	Months From Last Ablation to Detection
Koda et al, 2003^4^	IV, VI, VII, VIII	Pleural effusion progressing to large intestine	8 and 13
Nakamura et al, 2014^6^	IV, VIII	Small intestine	20
Liang et al, 2016^3^	VIII	None, pleural effusion	8
Kimura et al, 2021^5^	VI, VII	Small intestine	22
Hoskovec et al, 2023^7^	Not specified	Right liver lobe	15

## CASE REPORT

A 75-year-old female was on scheduled follow-up because of her medical history that included diverticulosis and ovarian adenocarcinoma. Surgical history included hysterectomy (in 1995, 23 years prior to presentation) and bilateral salpingo-oophorectomy with omentectomy, debulking, and lymphadenectomy (in 2015, 3 years prior to presentation). The patient underwent adjuvant chemotherapy. During scheduled surveillance 3 years after her surgical resection, the patient had an increase in CA-125 levels from a baseline of 5-7 units/mL to 17.4 units/mL (reference range, 0-35 units/mL). The patient also reported new right upper quadrant abdominal discomfort that had progressed during the prior 6 months.

In December 2018, computed tomography (CT) scan of the abdomen/pelvis revealed 2 hypodense liver lesions in segment VII (the first located in the cephalad portion of the segment along the dome and measuring 22 × 16 mm; the second located in the inferior/lateral portion of the segment and measuring 16 × 14 mm). The biopsy finding was poorly differentiated adenocarcinoma consistent with ovarian primary.

In February 2019, the patient underwent microwave ablation of the 2 segment VII lesions, with the procedure complicated by pneumothorax requiring a chest tube. Surveillance CT scan in April 2019 demonstrated continued growth from the segment VII dome lesion, prompting a second ablation in May 2019. At her 2-month follow-up appointment, the patient reported mild right upper quadrant discomfort that was improved from the prior report. Ongoing surveillance visits showed return to baseline of CA-125 and stable postablative changes without growth on CT scans in June, August, and October 2019.

**Figure 1.**
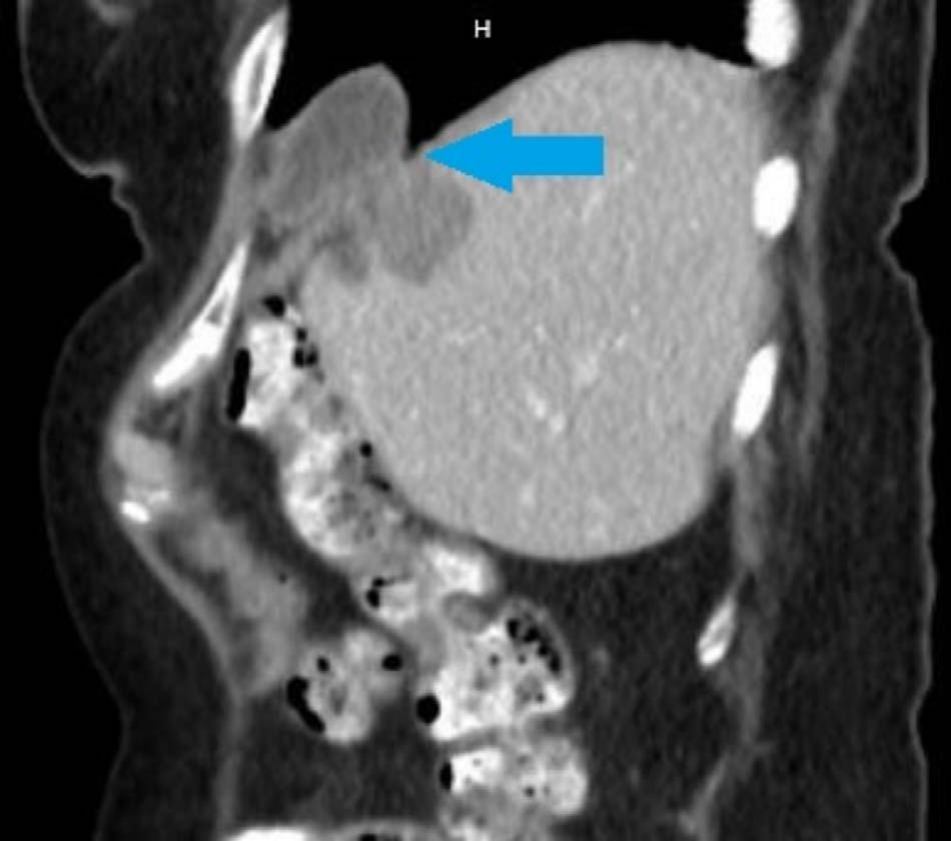
Computed tomography sagittal view shows gallbladder diaphragmatic herniation (arrow).

**Figure 2.**
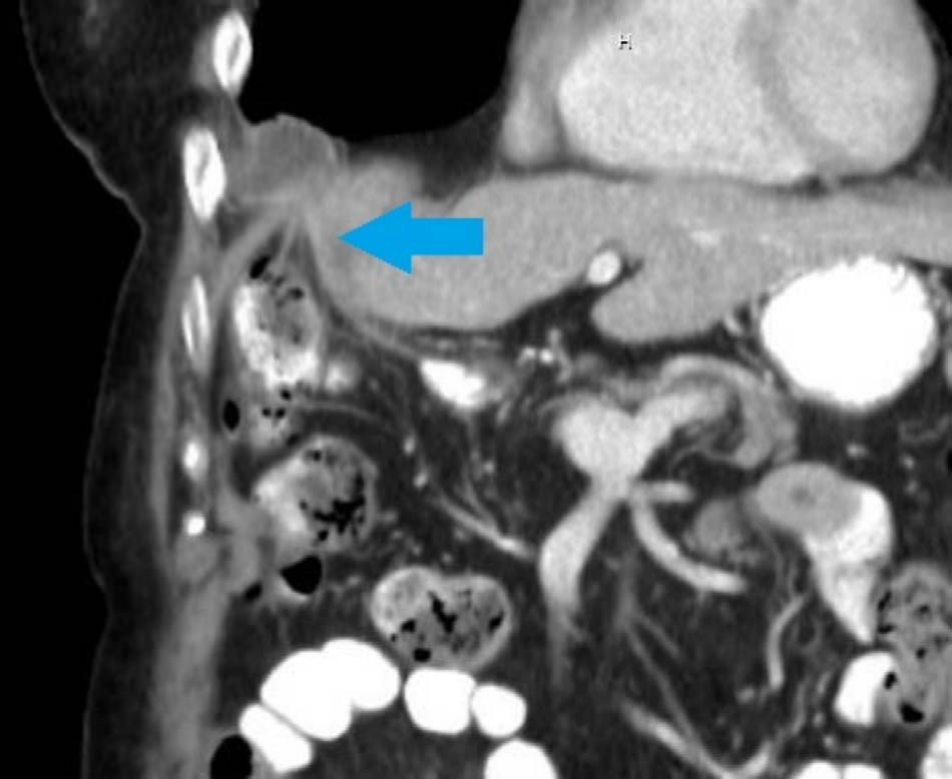
Computed tomography coronal view shows gallbladder diaphragmatic herniation with prominent cystic duct (arrow).

**Figure 3.**
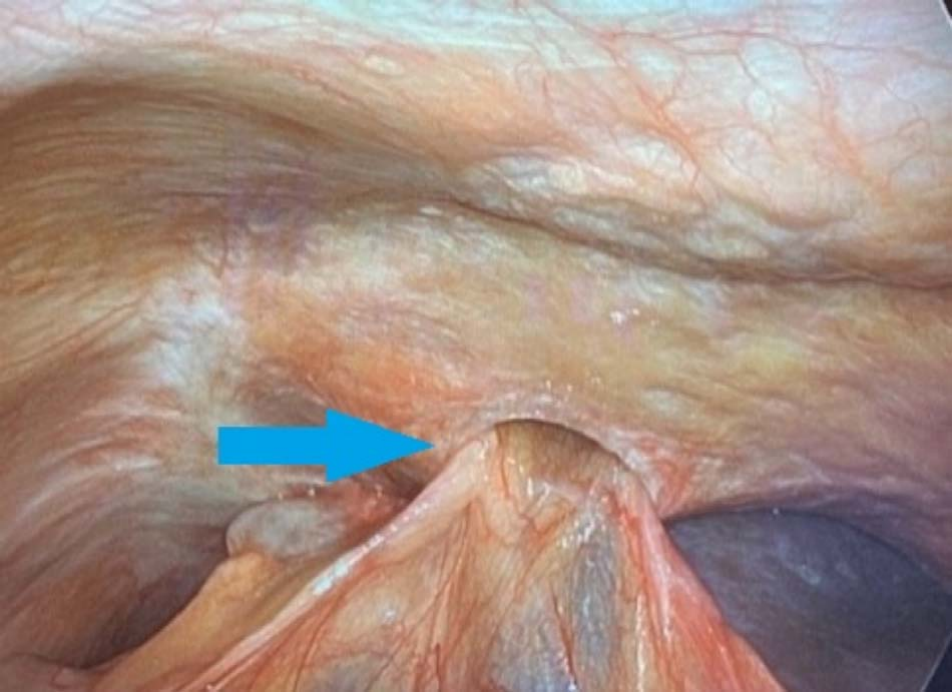
Intraoperative image shows the gallbladder herniating through the diaphragm (arrow).

**Figure 4.**
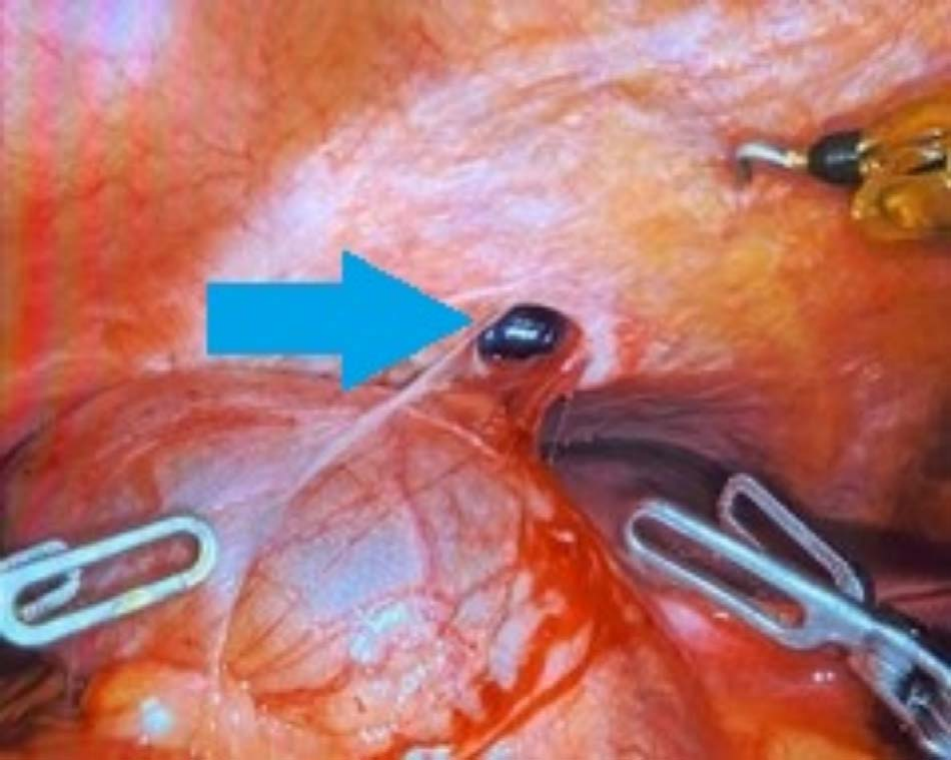
Intraoperative image shows the primary diaphragmatic defect (arrow) after reduction of the gallbladder.

**Figure 5.**
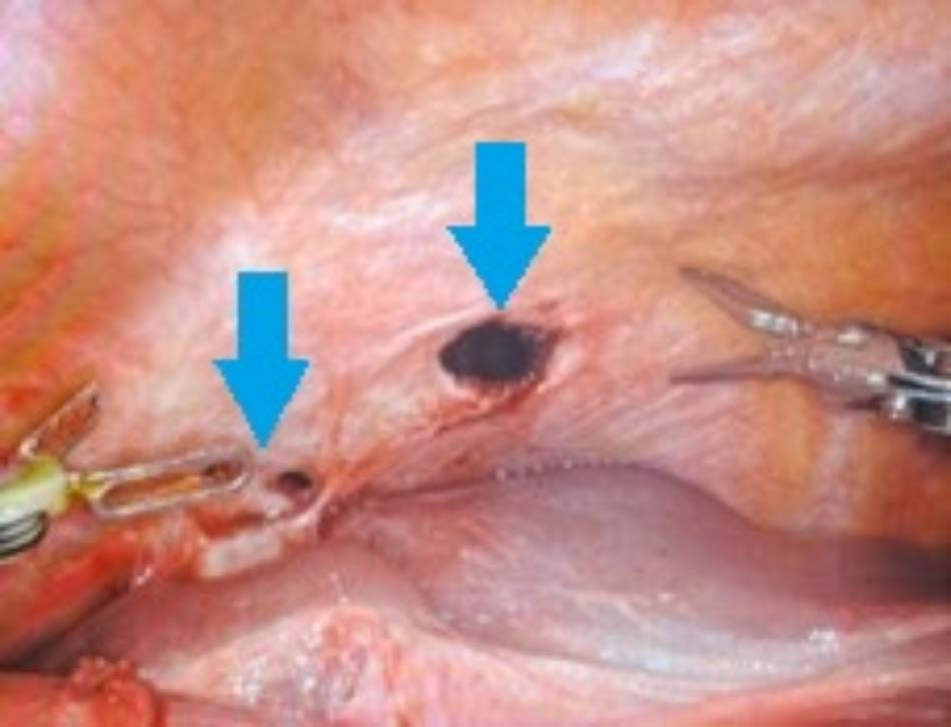
Intraoperative image shows diaphragmatic defects prior to repair (arrows).

**Figure 6.**
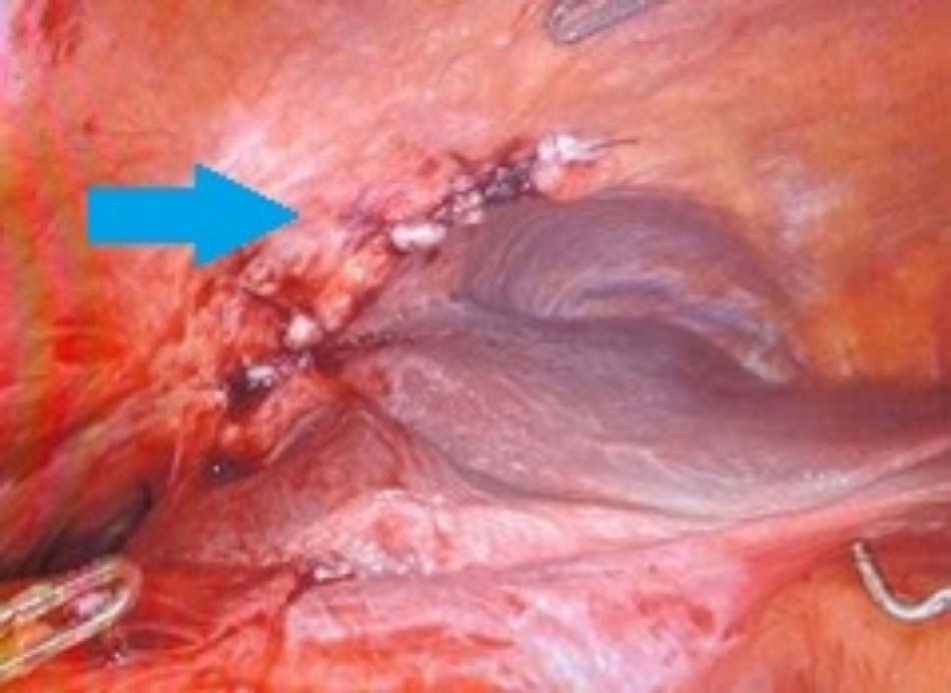
Intraoperative image shows the diaphragm after primary repair (arrow).

Two years after her ablation, the patient reported persistent and worsening right upper quadrant pain, right shoulder pain, and nausea, prompting a CT scan of the abdomen/pelvis in July 2021 that revealed a diaphragmatic hernia containing her gallbladder (Figure 1). Visualization of the cystic duct confirmed the diagnosis (Figure 2). The patient underwent robotic-assisted laparoscopic cholecystectomy with repair of the diaphragmatic defect. Some mild adhesions between the diaphragm and liver were encountered and lysed (Figure 3); however, the gallbladder was easily reduced (Figure 4), revealing 2 diaphragmatic defects (3 cm and <1 cm in size) (Figure 5). The diaphragmatic defects were repaired primarily prior to cholecystectomy (Figure 6). The gallbladder pathology was benign with cholesterol polyps and evidence of mild chronic inflammation.

At 3-week follow-up, the patient reported that she was recovering well, her pain had resolved, and she was tolerating diet.

## DISCUSSION

Our patient endorsed some mild right upper quadrant abdominal pain on 2-month follow-up after ablation but did not demonstrate severe symptoms for 2 years. Other cases of diaphragmatic hernia after ablation also had delayed presentations: pleural effusion at 8 months with herniation of the large intestine 13 months after ablation^4^ and herniation of the small intestine 22 months after ablation.^5^ These delays in onset of symptoms support the theory that small defects caused by traumatic or thermal injury can initially be subclinical, particularly in patients whose injury abuts the diaphragm as the defect can be protected by the presence of the liver. Over time, the size of the defect increases, and the patient can begin to have symptoms. Our patient may have had worsening biliary colic because of intermittent gallbladder herniation or mechanical obstruction of the cystic duct.

Case reports that describe diaphragmatic defects after liver ablation can help clinicians who are managing these patients. Suspicion for injury should be high in patients with a delayed presentation of symptoms. Early diaphragmatic defects can be detected with the presence of referred shoulder pain or pleural effusion. Ongoing delay in diagnosis can lead to organ herniation that can present with organ-specific findings. Clinicians should also be aware of the increased risk of diaphragmatic injury with ablation of tumors that abut the diaphragm (eg, liver segments VII/VIII).^6^

## CONCLUSION

Diaphragmatic hernia is a rare complication of hepatic lesion ablation, and this case demonstrates a unique complication of gallbladder herniation. Clinicians should be aware of this complication and note subtle symptoms such as small pleural effusions, right upper quadrant pain, and referred shoulder pain that may indicate an evolving diaphragmatic hernia. Early identification can be crucial to prevent severe complications and challenging surgical interventions.

## Notes

*This article meets the Accreditation Council for Graduate Medical Education and the American Board of Medical Specialties Maintenance of Certification competencies for Patient Care and Medical Knowledge.*
